# A grounded theory of clinical nurses’ process of coping during COVID‐19

**DOI:** 10.1111/jocn.15809

**Published:** 2021-05-06

**Authors:** Lorelli Nowell, Swati Dhingra, Kimberley Andrews, Jennifer Jackson

**Affiliations:** ^1^ Faculty of Nursing University of Calgary Calgary AB Canada

**Keywords:** coping, disaster, grounded theory, nurses, nursing workforce

## Abstract

**Aims and Objectives:**

To explore clinical nurses’ process of coping during COVID‐19 and develop a grounded theory that can be used by leaders to support clinical nurses during a disaster.

**Background:**

The COVID‐19 pandemic has provoked widespread disruption to clinical nurses’ work. It is important to understand clinical nurses’ processes of coping during disasters to support the nursing workforce during events such as global pandemics.

**Design:**

We employed the Corbin and Strauss variant of grounded theory methodology, informed by symbolic interactionism, and applied the EQUATOR guidelines for qualitative research publication (COREQ).

**Methods:**

Data collection entailed semi‐structured interviews with experienced clinical nurses (n =20) across diverse settings. We analysed data by identifying key points in the nurses’ coping processes inductively building concepts around these points.

**Results:**

The predictor of nurses’ outcomes in this grounded theory was their confidence in their ability to cope during the pandemic. When nurses lacked confidence, they experienced working in the context of *acute COVID*—a state of chaos and anxiety, with negative consequences for nurses. However, when nurses were confident in their abilities to cope with the pandemic, they experienced working in the context of *chronic COVID*, a calmer state of acceptance. There were many workplace factors that influenced nurses’ confidence, including adequacy of personal protective equipment, clear information and guidance, supportive leadership, teamwork and adequate staffing.

**Conclusions:**

Understanding clinical nurses’ experience of coping during COVID‐19 is essential to maintain the nursing workforce during similar disasters.

**Relevance to clinical practice:**

Nurse leaders can target areas that support nurses’ confidence, such as adequate PPE and staffing. In turn, increased confidence enables clinical nurses to cope during disasters such as a global pandemic.

**What does this paper contribute to the wider clinical community?**

- Nurses benefit from feeling confident about their ability to cope during disasters.
- This confidence is influenced by factors at work, notably, adequate supplies, clear guidance, leadership, collaboration and adequate staffing.
- Nurse leaders can support clinical nurses’ coping during disasters by applying this grounded theory and fostering nurses’ confidence.

## 1 INTRODUCTION

COVID‐19 has changed how nurses provide care worldwide. Nurses spend the most time with patients (Bridges et al., 2012; Lavander et al., 2016), meaning that nurses are exposed to significant challenges through their work in the pandemic. Prior to COVID‐19, nurses faced challenges related to burnout (Aiken et al., 2002; Epp, 2012; Jackson et al., 2018), maintaining staffing levels and retaining nurses (Aiken, 2002; Rafferty et al., 2007), and job dissatisfaction (Aeschbacher & Addor, 2018; Al Maqbali, 2015; Bamford & Hall, 2007). With the onset of COVID‐19, these challenges have likely heightened, as nurses are working tirelessly through this global disaster.

## 2 BACKGROUND

The COVID‐19 pandemic has highlighted gaps in healthcare systems and nursing support that are required during disasters (Daly et al., 2020). Previous studies have found that nurses are often ill‐prepared for working during disasters (Labrague et al., 2017). The lack of PPE and emotional distress are major concerns regarding the health and safety of nurses across the world (Daly et al., 2020). Given the high burden of COVID‐19, there is a growing demand to support nurses across the world to counter the potentially harmful consequences of working during the pandemic (Cavallo et al., 2020; Chen et al., 2020; Kang et al., 2020; Legido‐Quigley et al., 2020; Ran et al., 2020; Wang et al., 2020).

The clinical nurses working during the COVID‐19 pandemic need to know that their concerns are understood by nurse leaders (Daly et al., 2020). At present, there is a lack of evidence‐based frameworks on how to support nurses during this crisis. To support nurses adequately, policymakers and healthcare leaders must understand nurses’ needs during the pandemic and be responsive to these needs with meaningful support (Fernandez et al., 2020). The purpose of this study was to explore nurses’ process of coping during COVID‐19 and create a framework for supporting coping among clinical nurses.

## 3 METHODS

We used Corbin and Strauss (2014) grounded theory methodology to understand the process of clinical nurses coping during COVID‐19 and applied the EQUATOR guidelines for publication in qualitative research (COREQ) (Supplementary File 1). Grounded theory is an inductive method of creating a theory from data (Corbin & Strauss, 2014), where researchers foster critical reflection throughout the process by asking questions, seeking clarification and actively listening to participants’ stories. Researchers also draw on personal experiences to facilitate theoretical development, as active agents in the research process (Corbin & Strauss, 2014).

### 3.1 Symbolic interactionism

Our grounded theory methodology was rooted in symbolic interactionism (Blumer, 1969). The symbolic interactionism framework has three major assumptions: culture influences how people live and learn, experiences through culture determine how people make meaning from their interactions, and everyone creates meaning on an individual level and acts according to this meaning (Blumer, 1969). Therefore, understanding a phenomenon is specific to the context in which it was evaluated. The use of the grounded theory methodology inherently acknowledges that the context of a theory is inseparable from the theory itself (Milliken & Schreiber, 2012). In this study, the understanding of nurses’ experiences is based on the context of COVID‐19.

### 3.2 Context and researchers

The members of our all‐female research team have backgrounds in health care and nursing, with expertise in qualitative research. The research took place as the initial COVID‐19 peak and lockdown were occurring in Canada in spring/ summer 2020. There were no prior relationships with the participants, and participants were interviewed at various stages of the COVID‐19 pandemic in their respective countries. Participants were aware of the purpose of the study and were encouraged to share their views freely.

### 3.3 Sampling

We used purposive and convenience sampling strategies to recruit participants who worked clinically (Richards & Morse, 2013). Participants were invited to join an earlier phase of the study through social media posts, emails and word of mouth. Additionally, there was an element of theoretical sampling in the study (Richards & Morse, 2013). We recruited participants based on their responses to a survey in an earlier phase of the study, to support theoretical development. Survey respondents included nurses working in academic, community, management and clinical nursing roles. We purposively sought out nurses working in clinical nursing roles to explore their unique experiences and processes of coping during COVID‐19.

Participants indicated on our previous survey (Nowell, L. Dhingra, S. Andrews, K. & Jackson, J. ‘Perceptions and Nursing Demands and Experiences In the Middle of an International Crisis (PANDEMIC)’. International Nursing Review. Submitted) whether they would be willing to be contacted for an interview, by providing an email address. Participants had provided demographic information with the survey, and these data with their survey responses were used to recruit potential participants for this study. Potential participants received an email inviting them to complete an interview. There were three reminders sent as follow‐ups. A total of 45 participants were approached and 20 completed interviews.

### 3.4 Data collection

We collected data using a semi‐structured interview guide (Table 1). Two researchers (SD and KA) carried out the one‐on‐one interviews via zoom lasting 30–45 min. All interviews were audio‐recorded and transcribed verbatim.

**TABLE 1:** Semi‐structured interview guide

Interview questions
Can you tell me the story of how you became a nurse and what motivated you to choose this career path? What are you most proud of regarding your nursing work during COVID? Why? What has been the most challenging aspects of your nursing work during COVID? Why? Have you experienced any personal challenges at work or at home, or managing both? How has this affected you mentally and emotionally? How has COVID affected you socially? How well do you feel these issues have been addressed by your employers and leaders? Was there any sort of support offered by your organisation/employers? To what extent you feel any of these services you mentioned have been used? In your opinion, has this been a successful approach? Why or why not? What suggestions would you make for how these issues could be better addressed by the system? Do you feel that working during COVID has impacted you in any positive ways? Is there anything else you would like to share with me, or, hoped that I would have asked you?

### 3.5 Data analysis

As their variant of grounded theory has evolved, Corbin and Strauss (2014) placed less emphasis on specific, labelled levels of coding and advocated for an overall inductive process, which gradually refines the categories present within the data. We began analysis by having all the researchers read 2–3 interview transcripts and talk about our initial impressions. These discussions enabled reflexivity and collaboration on ideas. We recognised that there was a transition point for many participants where they had gone from an anxious stage of managing COVID‐19 to a calmer experience. Two researchers began by coding this transition point (or identifying participants who did not experience the transition) in NVIVO and building out from there. We repeated a process of reading interviews, discussing emerging ideas and further coding several times as we developed the theory. Applying the constant‐comparative method, the researchers compared each piece of data to all others for the duration of the study (Connelly, 2013). This is consistent with recommendations for all data to be given equal consideration during analysis (Morse, 1995). We continued coding and discussing our findings until we achieved a measure of saturation from our data.

### 3.6 Saturation

A hallmark of developing a grounded theory is achieving theoretical saturation (Corbin and Strauss 2014). Saturation occurs when interviews reveal no new information (Richards & Morse, 2013). However, this belief is tempered by the symbolic interactionism tenet that it is impossible to account for all factors that influence a phenomenon (Corbin and Strauss 2014). Thus, theoretical saturation is present when a researcher has established clear linkages between concepts in a comprehensive theory (Morse, 1995). In our study, we determined we had achieved saturation when we had predictive capacity in our theory and could chart the path of each participant based on the theoretical categories.

### 3.7 Ethics

We obtained institutional ethics approval for this study (REB20‐0633). Clinical nurses provided written consent prior to the interviews. Verbal consent was confirmed at the start of each interview.

### 3.8 Rigour

We used several techniques to maximise the trustworthiness of study findings. Team meetings provided a venue for reflexivity, debriefing and asking questions of our interpretations and stance (Morse, 2015). We maintained a detailed audit trail of all decisions (Carnevale, 2016), including a codebook, meeting minutes and file naming conventions. Teams of two researchers (SD and KA) coded each transcript, and decisions about theory development were vetted within the team (Morse, 2015). We returned to the raw data to further verify our results and ensure that our theory adequately reflected the participant voices (Morse, 2015).

## 4 RESULTS

Twenty clinical nurses participated in this study. Participant demographics are displayed in Table 2. All participants were white females, and the majority had bachelor's degrees (60%), were from Canada (65%) and had 5–9 years of clinical nursing experience (55%). While all participants indicated they worked as clinical nurses in acute care, only five participants specified their acute care specialty areas. Two participants specified they worked in emergency, one stated they worked in ambulatory care, one indicated they worked as a flight nurse, and one highlighted their work area of acute care haemodialysis.

**TABLE 2:** Participant demographics

Demographic	Demographic subcategories	*n*	%
Country	Canada	13	65
	USA	3	15
	UK	3	15
	Australia	1	5
Age	25–34 years	6	30
	35–44 years	5	25
	45–54 years	9	45
Years in current position	Less than 1 year	2	10
	1–4 years	5	25
	5–9 years	11	55
	10–14 years	1	5
	15–19 years	1	5
Education	Bachelor's degree in nursing	12	60
	Master's degree in nursing	3	15
	College diploma	1	5
	Others	4	20

### 4.1 Overview of theory

**FIGURE 1:**
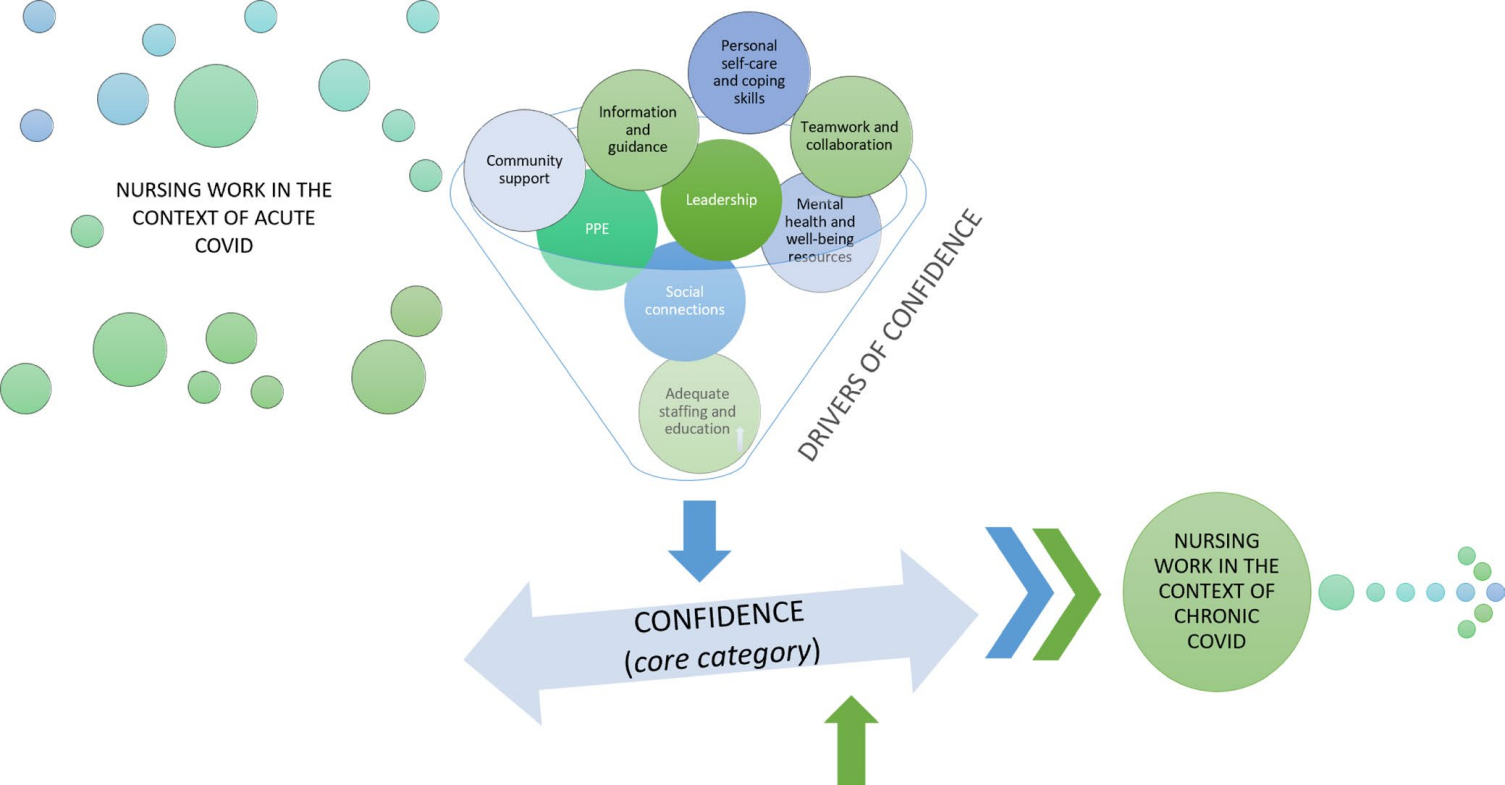
Clinical nurses’ process of coping during COVID‐19 [Colour figure can be viewed at wileyonlinelibrary.com]

The core category in this theory is confidence, which created the predictive capacity in this theory (Figure 1). When nurses were confident that they could manage the challenges from COVID‐19, they were able to move from working in the context of acute COVID, a phase of chaos and uncertainty, to working in the context of chronic COVID, a phase of acceptance and continuation. Without confidence, nurses remained in the acute phase. Nurses’ confidence was impacted by drivers that included clear guidelines, leadership, and adequate supplies and staffing. When there were few drivers, nurses lacked confidence in their abilities to manage and struggled. In turn, if there was enough influence from drivers to support nurses’ confidence in their abilities to manage, they could transition to a less chaotic phase and cope more effectively. These elements of the theory are explained further in the following sections.

### 4.2 Working in the context of acute and chronic COVID

COVID‐19 was the dominant contextual factor in this study. Participants lived in a state of ‘total COVID’, where the pandemic impacted every aspect of their lives. Even within the dominant context of COVID‐19, the pandemic was experienced in largely two different ways, termed working in the context of acute and chronic COVID‐19. These are explained in the following section.

#### 4.2.1 Working in the context of acute COVID

Working in the context of acute COVID occurred largely during the initial phase of the pandemic where there were still many unknowns and a lack of understanding of the disease. Nurses were challenged by the uncertainty. Many nurses described the impact of the constant changes, conflicting information and lack of evidence to support best practices.

I think in the first few months, because we didn't have as much data, and we were working obviously with less information, we didn't know how infectious COVID was, we didn't know aerosolized versus airborne… you'd get conflicting information and colleagues would have really strong feelings they were supposed to be doing something one way that conflicted with our guidance from our leadership. (P18).

For some participants, this lack of information resulted in a lack of confidence in how to provide the best nursing care.

Working in the context of the acute phase of COVID was also characterised by uncertainty around supplies, which caused a great deal of distress. The lack of PPE was concerning for participants, especially when they were unsure whether they would have enough masks: ‘At the time we didn't know if we're going to have enough PPE, if we're going to have to ration it or reuse it…’ (P2). Overall, working in the context of the acute phase of COVID resulted in nurses having a lack of trust in information and supplies that made their jobs even more difficult.

Working in the context of the acute COVID phase resulted in fear and anxiety among participants. Nurses described feeling overwhelmed, anxious and nervous all the time, which was something they were not used to.

I noticed that there was a heightened sense of anxiety, there was a sense of fear. People didn't know what the new policies were going to be and the new practices. Every day we came to work, we were getting new best practices… people were very anxious…they would be completely scared that there was something that changed that they didn't know. (P14).

Acute COVID was very emotionally taxing. Participants feared exposure to COVID and being required to take on additional risk through their work. The acute COVID experience was ultimately a state that nurses experienced, rather than a discrete time frame. The anxious, chaotic and uncertain times in the pandemic constituted the acute COVID phase for participants.

#### 4.2.2 Working in the context of chronic COVID‐19

In contrast to working in the context of acute COVID, working in the context of chronic COVID occurred when nurses were confident in their abilities to manage during the pandemic. Participants reported feeling less uncertainty and that they were able to work without undue stress. ‘We've got all these systems in place, protocols, we've got all this experience now…we improved a lot’. (P1). Once nurses were confident there were adequate supplies, they felt better able to cope. There was also a sense of acceptance that COVID was not going away: ‘COVID is currently going to remain a part of our lives… I think what most people are doing to maintain sanity is become not complacent, but resigned to where we're at right now’. (P15). Nurses also expressed pride in the way they were able to come together with their colleagues and support each other throughout the pandemic: ‘You look back, and you think, "Gosh we did that." And that is just really quite nice to see, that actually can be pulled off and pulled together. Something that could be so complex’. (P17). These nurses had reached a phase of the process that was calmer and enabled them to have a higher level of functioning than when working in the context of acute COVID.

Some participants described a decrease in anxiety because they felt like they had what they needed to handle to complex challenges presented by COVID‐19. One participant stated ‘You relax in a different way, but it's not relaxing like it used to be pre‐COVID’. (P17). This participant recognised that they were still facing significant challenges, but not with the same anxious energy as working in the context of the acute COVID phase. Participants reported settling into the new processes and being better able to adapt to changes. Another participant described it as having a new routine quality: ‘Now that we've gotten into a groove and we have enough equipment, we have enough PPE, we have multiple contingency plans in place for over capacity. I feel much better’. (P2). Participants reported that they were calmer, better able to plan and had increased trust that they would be able to manage changes in the status of the pandemic.

### 4.3 Core category: confidence

The core category in this grounded theory is confidence. When participants gained increased confidence in their abilities to manage during the pandemic, they transitioned from working in the context of acute COVID to chronic COVID. This transition was fostered by various drivers, which are described in the following section.

Participants reported confidence as a series of personal and professional realisations, which made them feel more positive, hopeful and self‐assured to keep working through the pandemic. Participants reported increased confidence when they had adequate supports and supplies to manage during the pandemic and that they could trust that the resources they needed would be available. Some participants identified confidence as finding the fortitude and inner strength to keep moving forward. This participant explained the role of confidence in transitioning to working in the context of the chronic COVID phase:

I think after I had that first panic, anxiety reaction, I suppose, I just looked at the situation at hand and the recognition of we still have to be able to move forward in life even when there are things going on that are definitely fear‐inducing. If you can't move past that, you've defined the way that you're going to be living your life and I don't want to live in a fearful manner […] changing the narrative from fear to logic helps me to get through the day‐to‐day because I feel that that allows me a sense of control. (P15).

Confidence helped participants to understand and adapt to the evolving pandemic situation. This participant reported that as her confidence increased: ‘my anxiety started to decrease because I felt we could handle it’. (P13). These participants reported a qualitative difference between working in the context of the acute and chronic COVID phases, which impacted their process of coping.

Predictive capacity was demonstrated in this grounded theory through the core category of confidence, in part through examples from negative cases. While most participants reported spending time working in the context of acute COVID, there were others who did not experience that anxiety. These nurses were confident about their abilities to manage from the beginning of the pandemic and thus entered the process at working in the context of chronic COVID phase. These participants were less impacted by the uncertainty, despite the challenges and stresses of working in the context of acute COVID. This confidence was most often related to previous work experiences with similar types of situations.

Working 30 years, I've been through a few things. I've worked through SARS. I worked through the H1 N1… I worked through when AIDS was very new… because I have worked a long time and I've worked through other sort of intense periods, I was picking up sort of the anxiety of my coworkers too… I was thinking, am I missing something? Should I be more anxious about this? Am I too laid back? (P10).

The nurses who started the process of working in the context of chronic COVID phase began the pandemic with the confidence that they would be able to cope, whatever happened. This participant placed the experience in context: ‘just something that we're going to have to deal with, people before us have dealt with a lot worse than what this is’. (P7). These participants did not feel undue anxiety, because they reflected on personal experiences and historical examples, and had confidence that they would get through COVID‐19. These findings demonstrate that confidence was the predictive factor that made the difference in participants’ experiences of working in the context of either acute or chronic COVID.

Additionally, some participants stayed in working in the context of acute COVID phase throughout the pandemic, because they never gained sufficient confidence to transition. These examples further illustrated the predictive capacity inherent in confidence. Some participants felt mentally and emotionally drained, to the point where they were ready to leave nursing altogether.

The staff are just exhausted, like really, exhausted. Because they've been through months of this huge level of stress, and their lives have been very difficult at home and their work has been really challenging. And so, there's a lot of fatigue. (P1).

Other participants spoke about the consequences for them of this continued anxiety, including sleepless nights and week‐long migraines. Their worry about the pandemic and its potential impact on their families was all‐consuming.

Emotionally, I'm always worried. Am I going to bring home COVID to my family? Am I going to infect my father‐in‐law who has a heart condition? If I go anywhere, am I going to be the one who spreads it at the grocery store, even though I'm always wearing a mask? It's always just a worry and a risk. (P6).

This participant demonstrated that even after time had passed since the start of the pandemic, they were still feeling heightened anxiety and burnout. These participants had not reached adequate confidence to move through the process to work in the context of chronic COVID and thus faced emotional exhaustion. Confidence was influenced by multiple drivers, which are explained in the next section.

### 4.4 Drivers

There were several drivers for clinical nurses that helped or hindered their confidence in their ability to work during the pandemic. These drivers included factors at work and factors outside of work. Nurses had varying degrees of control over these factors and could be negatively influenced by poor working conditions. For example, many participants described symptoms of burnout related to working mandatory overtime, managing heavy workloads and constant changes in COVID policies. The presence or absence of these drivers served to reinforce or undermine nurses’ confidence in their abilities to manage during the pandemic. The drivers of confidence at work offered nurses little personal control but were an important part of developing nurses’ confidence. The contribution of the drivers to the process of coping during the pandemic is discussed in the following section.

#### 4.4.1 Drivers of confidence at work

There were several drivers of nurses’ confidence at work, which included adequacy of PPE, clear guidance, leadership, teamwork and adequate staffing. These are discussed below.

##### Personal protective equipment (PPE)

Participants reported that appropriate PPE was needed to ensure the safety and well‐being of clinical nurses, patients and everyone in clinical settings. Several participants highlighted the stress related to insufficient PPE.

Normally of course we have as much PPE as needed and having to be very careful about the PPE we're using and not knowing if we were going to have it in the future was a huge burden and a huge challenge for a lot of us. (P13)

This participant explained that prior to COVID‐19, nurses had always known that they had sufficient PPE and could protect themselves from pathogens. When nurses no longer had this certainty, their confidence decreased. Conversely, when the supply of PPE was adequate, participants reported increased confidence.

Once we got into the swing of things with our PPE and everything, we all felt quite confident because none of us had gotten sick and none of us have contracted it. So we all felt confident in our PPE, So, just knowing that now that we have enough and we have all these plans in place for extra supplies, I feel good. (P2).

This participant demonstrated how the adequate PPE inspired confidence among nurses because they knew they could work more safely in environments with COVID‐19. PPE also signalled to nurses that their organisation could manage the crisis and maintain supply chains, which fostered confidence by using PPE as a proxy.

##### Information and guidance

Nurses reported that they needed clear and transparent communication regarding the best practices and guidelines to work safely during COVID‐19. Participants indicated that having a resource person to provide regular updates with appropriate guidance on the changing policies was helpful. The frequency of updates made it hard to keep track, and the resource person helped to clarify the current best practices.

Their job [resource person] was to know what the latest news was. So even if we weren't up‐to‐date they were, so we could always go and talk to them which was very helpful… so we could focus on the patient care and they could focus on the politics, the rules, all that stuff. (P2).

Paradoxically, some participants mentioned that too much information, or irrelevant information, added unnecessary stress: ‘At the beginning I read so much information. I was very overwhelmed. I was super anxious. I was super nervous all the time’. (P11). Conflicting, contradicting and/or inadequate information intensified the challenges that many nurses experienced. Participants required a balance of not only knowing the latest information to guide their work, but also avoiding an overwhelming deluge of material.

##### Leadership

Participants indicated that trustworthy leadership and guidance was an important factor that helped them cope with the pandemic's unpredictability. Supportive leadership behaviours were appreciated by several participants who stated that the opportunity to voice their concerns and receive support from their leaders supported their confidence: ‘I felt like they were available and that they would do their best to provide resources if we needed them…their leadership behaviours were very supportive’. (P18).

In contrast, some participants noted untrustworthy leadership and poor guidance precipitated their anxiety and undermined their confidence. Additionally, the perceived disconnect between clinical nurses and decision‐makers made participants feel less confident that they would have adequate support. ‘The decision‐makers seemed to be not on the front lines. So there were issues that would come up pragmatically that they wouldn't understand and that was a little concerning’. (P14). When their leaders appeared disconnected from clinical challenges, participants felt that they may not have the necessary support to work safely during the pandemic. In turn, this undermined confidence and contributed to nurses remaining to work in the context of acute COVID phase.

##### Teamwork and collaboration

Working with a collaborative team and having shared decision‐making opportunities supported the confidence of several participants. The teamwork created solidarity, which helped nurses to feel more confident in their work. This participant also explained how shared decision‐making created a sense of control in a chaotic context.

I'm lucky that I work with a collaborative team. And so a lot of our guidance from our co‐workers … of a lot of shared decision‐making and working together to kind of manage risk as best as we can to keep everyone safe. (P5).

This participant was confident in their safety and that of their colleagues, which enabled them to cope effectively in challenging conditions. As one participant noted, when there was a lack of collaborative approaches, it strained nurses’ confidence. ‘It was all quite disjointed and vague and like no one really knew what was going on’. (P8). This participant did not experience a sense of solidarity, which created a lack of confidence. This increased the participants’ anxiety, preventing nurses from moving to work in the context of chronic COVID phase.

##### Adequate staffing and education

Participants identified that having adequate staffing with clear role descriptions and sufficient education available supported their confidence. In contrast, insufficient education, especially in cases of redeployment, contributed to increased stress and anxiety among participants.

When I was asked to go onto the wards, the expectations were just impossible for me to achieve what I needed to be able to do. They felt that I could move my role and still work at the level of my banding. So as a senior nurse, I was expected to act as a senior nurse on the oncology ward, which eventually, I actually was unable to. I actually struggled even getting out of the car to get to work. I wasn't sleeping. This all happened very quickly, within a week. (P3).

This participant reported that they felt pushed into a new role with insufficient support and preparation. The role was also outside their expertise, which undermined the nurses’ confidence. The lack of familiarity and local knowledge was a notable barrier for participants. Additionally, participants reported that there were not enough staff in general to keep up with the demands, regardless of redeployment. Inadequate staff to patient ratios further reduced the ability of some participants to cope with the work demands.

We have too many patients for the actual available personnel to deal with. I wouldn't be so stressed out if I didn't have six patients every single shift…if I have an additional nurse on my shift, I don't have that stress. (P6).

This participant reflected that they needed additional help because the high ratio of patients to each nurse created an unsafe situation. The stress of not having enough staff or not having the right preparation impeded nurses’ confidence in their ability to work safely. In addition to these factors that impacted nurses’ confidence at work, they were also influenced by factors outside of work. These are presented in the following section.

#### 4.4.2 Drivers of confidence outside of work

In addition to workplace drivers, participants reported that their confidence was influenced by support from their communities, personal coping strategies, mental health and well‐being resources, and social connections. Each is presented below.

##### Support from community, family and friends

Participants highlighted how support from the community, family and colleagues was essential to their overall coping with work demands during COVID. Support from the local community helped boost morale and provided motivation to keep going.

We had meals brought in, we had notes, we still have little signs placed all over the hospital like in the parking lot about: You're heroes and you can do this and we're, we're, we're behind you and those kinds of sentiments and all various forms. And then my neighbours put a sign in my yard and brought me a goody basket when they found out that I was a frontline worker … We didn't ask for that recognition, we just went to work as usual but those sort of things were happening all around. (P19).

This groundswell of support reinforced participants’ confidence, because it demonstrated the faith that the community had in nurses’ abilities to manage during the pandemic. For other participants, the absence of any such support hindered their confidence and motivation. A participant felt undervalued, which had a discouraging impact on their confidence. ‘we were not valued as humans and skilled workers but just cogs in the wheel […] that was a lot of source of stress for me’. (P13). For this participant, a lack of external support undermined their confidence, rather than other participants who said that community support reinforced their confidence.

##### Personal self‐care and coping skills

Participants mentioned that having healthy coping mechanisms and practising self‐care impacted their confidence in their ability to deal with the stress related to the pandemic. ‘I'm just happy that I have healthy coping mechanisms because it has been an isolating experience…’ (P14). This participant recognised the value of personal strategies that could be applied in the pandemic context. While some of the participants mentioned the use of their regular self‐care techniques such as meditation and yoga, others noted how COVID impaired their usual self‐care strategies. This participant recognised that they were struggling when they had physical changes such as sleep disturbances.

So certainly for the first, the weeks of changing role, I wasn't sleeping. I was over eating, continue to overeat, really. I've put on a lot of weight to comfort eating. Yes, I also had, in that first week, I had a feeling that I couldn't take any information in at all. So on top of trying to do… I couldn't take any information in. (P3).

This participant recognised that their coping strategies were not necessarily effective, but that these strategies were also a response to an overwhelming situation. Participants who engaged in positive coping strategies appeared to have an increased confidence in their ability to work through the pandemic, while others struggled.

##### Mental health and well‐being resources

Nurses reported that the availability of mental health and well‐being resources, including opportunities to debrief and adequate time away from work, had a positive impact on their COVID management response. Participants stated that having access to counselling services and debriefing sessions was successful approaches in managing work‐related stress and anxiety.

Just to offload everything was quite… Was really helpful, and hearing other people that had felt the same about things, it was reassuring in a way. That we'd all felt the same and that they were… Because we were all saying the same thing as well. (P16).

This participant indicated that the opportunity for debriefing relieved the burden of their stress and anxiety, and restored their confidence and solidarity with colleagues.

On the contrary, barriers such as a lack of knowledge on how to access mental health resources, a lack of opportunities to debrief and inadequate time off contributed negatively to the confidence of participants.

I haven't had any management or leader approach asking about coping or stress or anxiety or anything at home. We did get the healthcare worker bonus, I would say that's an acknowledgement to the work that we've put in. But no, there hasn't been any extra mental health support or emotional support. (P14).

This participant recognised that they did not receive support in an area where it would have been valuable. This participant also demonstrated that the additional payment was encouraging, but was not sufficient to offset a lack of emotional support. For some of the participants, the mere existence of support services was not helpful and indicated there were barriers to accessing the services.

##### Social connections

Participants stated that maintaining social connections was a source of support that reinforced their confidence. Maintaining social connections also contributed to knowledge sharing and helped participants learn from the experiences of their peers.

Through social media, you have networks of professionals and friends…from all over the nation and we would have Zoom meetings and these little happy hours and stuff and be like, "Hey, are you guys seeing this yet? What are you seeing? Has it come to your hospital yet or your state or your city yet? Are you seeing cases? What are you guys doing? What's your hospital protocol?" We would just compare notes. (P19).

The comradery that these connections created helped participants to have a broader view of COVID. Both the practical advice and the emotional support from social connections helped participants to build their confidence.

Over time, these drivers had the combined effect of increasing or decreasing a nurses’ confidence. While the influence of individual drivers was unique to each participant, they had the net effect of instilling a participant with a sense of either I can do this or I cannot do this. This confidence was integral to nurses moving to a calmer phase of working in the context of chronic COVID or staying in the anxious chaos phase of working in the context of acute COVID. Nurses do have an element of control over factors such as personal self‐care and seeking support. However, it is important to recognise that nurses’ environments are extremely influential, and the responsibility to cope well during the pandemic cannot be ascribed to individual nurses’ coping strategies.

## 5 DISCUSSION

In this study, we used Corbin and Strauss (2014) grounded theory methodology to understand the process of clinical nurses coping during COVID‐19. Our main finding was nurses’ confidence in their abilities to cope during the pandemic predicts whether they will experience working in the context of acute or chronic COVID. Nurses reported both drivers at work and outside of work that impacted their confidence. These drivers are key areas to provide support to clinical nurses to help them cope during disasters such as a global pandemic. This study adds to the literature by providing a consolidation of findings from prior studies and illustrating nurses’ process of coping during the pandemic.

Concerns regarding the lack of PPE and emergency reserve medical supplies were a central concern for many participants in this study. This stressor manifested as fear or anxiety of running out of supplies and becoming infected or infecting family. The lack of PPE has been identified as a major physical health stressor among healthcare workers during pandemics (Fernandez et al., 2020; Shaukat et al., 2020). It is important to secure PPE supplies to ensure nurses can be confident in providing competent care while maintaining their own health and safety.

Information and guidance were reported to change with a rapid pace that contributed to stress and a lack of confidence to provide safe patient care. Due to the novel nature of this virus, many have reported on increased confusion regarding the most up‐to‐date information (Fernandez et al., 2020; Liu et al., 2020). Establishing a resource person or structured pathways to disseminate information in a format that is easy to understand and follow may reduce anxiety and improve the nurses’ confidence in the care that they are able to provide.

There have been many studies exploring the impact of leadership and resources on nurses’ outcomes that reinforce our study findings. There is consistent evidence that adequate staffing and supportive leadership are key considerations in patient and nurse outcomes (Aiken et al., 2012; Kieft et al., 2014; Swiger et al., 2017). Others have highlighted the importance of leadership and guidance to support nurses work during COVID‐19 (Burch, 2020; Turale et al., 2020) and to help manage the uncertainty and anxiety associated with this pandemic (Fernandez et al., 2020; Mo et al., 2020; Zhang, 2020). Strong nurse leadership helps support the nursing workforce to better serve the patients, families and communities (Daly et al., 2020).

In our study, we found collaboration and teamwork helped increase nurses’ confidence in their ability to provide effective patient care. The importance of teamwork and collaboration during COVID has been discussed in the literature (Shinners & Cosme, 2020; Spoorthy et al., 2020). Nurses reported a sense of sharing the workload with colleagues, who have a personal understanding of the struggles and challenges that they face. These shared experiences offer a sense of support (Fernandez et al., 2020; Shih et al., 2007). For effective care to be delivered, trust and respect must be present among all members of the team, especially in highly uncertain times such as disasters or pandemics (Fernandez et al., 2020).

The emotional and psychological effects of working during COVID‐19 have impacted the way that nurses cope (Liu et al., 2020). The ability to gain support through social connections has drastically changed, as face‐to‐face or physical contact has been replaced with virtual connections. Nurses in this study have reported that social connections have had a positive effect on their ability to cope with the pandemic. Social support has been found to reduce stress, anxiety and feelings of isolation (Liu et al., 2020; Spoorthy et al., 2020; Xiao et al., 2020). Nurse leaders and organisations can promote social connections of nurses by establishing and fostering work‐related social connections (Liu et al., 2020; Zhang et al., 2020).

There have been multiple studies that have examined the psychological effects of work during the COVID‐19 pandemic (Liu et al., 2020; Spoorthy et al., 2020; Zhang et al., 2020), which conclude that there is a need for the provision of mental health services to address the mental well‐being of nurses. Likewise, the participants in this study reported positive effects on their overall mental well‐being when they were provided with mental health resources.

Engaging in personal self‐care is a strategy that allows nurses to increase confidence and reduce feelings of exhaustion and emotional depletion (Blake et al., 2020; Halcomb et al., 2020; Zhang et al., 2020). Nurse leaders can work to promote self‐care in the workplace by ensuring time is available for breaks, hydration and nutrition (Blake et al., 2020; Halcomb et al., 2020) and scheduling in a manner that prevents working excessive hours (Halcomb et al., 2020; Liu et al., 2020). Allowing for adequate self‐care in the workplace is crucial, as nurses often neglect their own health and well‐being out of a sense of responsibility to their patients (Liu et al., 2020). It is also important to highlight that nurses’ self‐care is an important consideration, but it does not replace the need for workplace supports such as adequate PPE and staffing.

### 5.1 Strengths and limitations

We used grounded theory methodology to understand the in‐depth perspectives of 20 clinical nurses who were working clinically during COVID‐19. We interviewed nurses from various countries and clinical settings during peak aspects of the first wave of the pandemic, and this provided valuable information about their experiences. However, the pandemic has continued to grow and evolve since the participants in this study were interviewed. While the drivers identified during the first phase of the pandemic are likely still relevant, there may be new concerns that might be important to consider. A further limitation of this study is that all participants were working in high‐income countries and may represent different experiences than nurses who live and work in developing nations.

### 5.2 Areas for future research

This study highlights key areas for future research including exploring interventions and strategies that can help address the drivers identified in this study. While there have been numerous interventions to improve work environments, it is unknown whether implementing these in the context of COVID requires different strategies. Though we have identified ways to support nurses’ confidence in their abilities to manage during the pandemic, we know less about how to maintain that confidence through long‐term chronic phases, which is a worthy area of further investigation.

## 6 CONCLUSION

Nurses’ contributions are significant in the delivery of care within the healthcare system. As clinical nurses working during COVID‐19 are experiencing additional stress and psychological vulnerability, it is important they have the appropriate supports to cope with this disaster, in order to maintain the nursing workforce. This study provides a theoretical framework that has the potential to help nurse leaders develop supports to allow clinical nurses to cope with disasters when applied in clinical practice.

### 6.1 Implications for nursing practice

There were many drivers in these findings that are in the remit of healthcare leaders, rather than individual clinical nurses. The workplace drivers were more influential in developing or undermining nurses’ confidence. Nurse leaders can use this grounded theory as a framework for supporting clinical nurses. These drivers provide guidance that informs support strategies for nurses working during a disaster.

## CONFLICTS OF INTEREST

The authors have no conflicts of interest to declare.

## Supporting information

Supplementary Material

## Data Availability

The data that support the findings of this study are available from the corresponding author upon reasonable request.
